# Evaluating the identification of the extent of gastric cancer by over-1000 nm near-infrared hyperspectral imaging using surgical specimens

**DOI:** 10.1117/1.JBO.28.8.086001

**Published:** 2023-08-22

**Authors:** Tomohiro Mitsui, Akino Mori, Toshihiro Takamatsu, Tomohiro Kadota, Konosuke Sato, Ryodai Fukushima, Kyohei Okubo, Masakazu Umezawa, Hiroshi Takemura, Hideo Yokota, Takeshi Kuwata, Takahiro Kinoshita, Hiroaki Ikematsu, Tomonori Yano, Shin Maeda, Kohei Soga

**Affiliations:** a National Cancer Center Hospital East, Department of Gastroenterology and Endoscopy, Kashiwa, Japan; b Yokohama City University Graduate School of Medicine, Department of Gastroenterology, Yokohama, Japan; c Tokyo University of Science, Department of Materials Science and Technology, Tokyo, Japan; d National Cancer Center, Exploratory Oncology Research and Clinical Trial Center, Chiba, Japan; e Tokyo University of Science, Research Institute for Biomedical Sciences, Chiba, Japan; f Tokyo University of Science, Department of Mechanical Engineering, Chiba, Japan; g RIKEN Center for Advanced Photonics, Saitama, Japan; h National Cancer Center Hospital East, Department of Pathology and Clinical Laboratories, Kashiwa, Japan; i National Cancer Center Hospital East, Department of Gastric Surgery, Chiba, Japan

**Keywords:** gastric cancer, near-infrared, hyperspectral imaging, tumor thickness, machine learning

## Abstract

**Significance:**

Determining the extent of gastric cancer (GC) is necessary for evaluating the gastrectomy margin for GC. Additionally, determining the extent of the GC that is not exposed to the mucosal surface remains difficult. However, near-infrared (NIR) can penetrate mucosal tissues highly efficiently.

**Aim:**

We investigated the ability of near-infrared hyperspectral imaging (NIR-HSI) to identify GC areas, including exposed and unexposed using surgical specimens, and explored the identifiable characteristics of the GC.

**Approach:**

Our study examined 10 patients with diagnosed GC who underwent surgery between 2020 and 2021. Specimen images were captured using NIR-HSI. For the specimens, the exposed area was defined as an area wherein the cancer was exposed on the surface, the unexposed area as an area wherein the cancer was present although the surface was covered by normal tissue, and the normal area as an area wherein the cancer was absent. We estimated the GC (including the exposed and unexposed areas) and normal areas using a support vector machine, which is a machine-learning method for classification. The prediction accuracy of the GC region in every area and normal region was evaluated. Additionally, the tumor thicknesses of the GC were pathologically measured, and their differences in identifiable and unidentifiable areas were compared using NIR-HSI.

**Results:**

The average prediction accuracy of the GC regions combined with both areas was 77.2%; with exposed and unexposed areas was 79.7% and 68.5%, respectively; and with normal regions was 79.7%. Additionally, the areas identified as cancerous had a tumor thickness of >2  mm.

**Conclusions:**

NIR-HSI identified the GC regions with high rates. As a feature, the exposed and unexposed areas with tumor thicknesses of >2  mm were identified using NIR-HSI.

## 1 Introduction

Gastric cancer (GC) is a commonly diagnosed cancer worldwide and one of the leading causes of cancer-related mortality.^1^ Gastrectomy is a curative treatment for GC.^2^ The effect of gastric remnant volume on the postoperative quality of life and nutritional status has been recognized. Furthermore, organ-sparing surgery is increasingly preferred.^3^^–^^5^ Japanese GC treatment guidelines (5th edition) recommend a proximal resection margin of at least 3 cm for tumors ≥T2 in the case of an expansive growth pattern, whereas a proximal resection margin of at least 5 cm is required in the case of an infiltrative growth pattern.^6^ The large margin in the infiltrative growth patterns is attributed to the extensive submucosal or deeper layer invasions. Hence, if the extent of the non-exposed cancer invasion is identified, the stomach can be resected more appropriately. However, assessing the extent of the tumor and determining the appropriate resection margins during surgery remains difficult. Hence, several methods, including intraoperative endoscopy and endoscopic tattooing or clipping, that can identify the tumor extent and determine the appropriate surgical margins exist.^7^^–^^10^ However, the endoscopic determination of the extent of the GC that is not exposed to the mucosal surface remains difficult (e.g., the infiltrative growth pattern^10^).

We previously reported that near-infrared (NIR) hyperspectral imaging (HSI) could be used to identify gastrointestinal stromal tumors in the muscular layer that was not exposed to the mucosa.^11^^,^^12^ Light in the NIR region, ranging from 800 to 2500 nm, is useful for probing deep parts of the tissues owing to its low absorption and scattering. The absorption spectra in the NIR region convey fingerprint data owing to the overtone or combined vibrations of the chemical bonds.^13^^–^^15^ This permits the investigation of the distribution of the chemical composition within a sample.^16^ Hyperspectral imaging provides a three-dimensional dataset (two spatial and one spectral) that allows a spectral curve at each pixel in the acquired images to be obtained.^17^ It uses a machine-learning algorithm to acquire spectral information from each pixel and extract critical imaging data from several hyperspectral images.^18^^,^^19^ Hyperspectral imaging has the potential for non-invasive, label-free diagnosis and surgical guidance. Hence, NIR-HSI can identify the extent of the GC that is not exposed to the mucosal surface.

Studies have reported the identification of GC using NIR-HSI, although its characteristics have not been considered.^19^^–^^21^ In this study, we aim to explore whether NIR-HSI can identify the GC regions, including each exposed and unexposed area, using surgical specimens and investigate the characteristics of the identifiable GC region.

## 2 Materials and Methods

### 2.1 Patients and Surgical Specimen

This study included 10 patients with clinically diagnosed GC who underwent surgery between September 2020 and October 2021. The inclusion criteria were as follows: (i) clinical diagnosis of the GC, (ii) age≥20 years, and (iii) written informed consent. The exclusion criteria were as follows: (i) history of prior chemotherapy and (ii) presence of the hepatitis B virus surface antigen or hepatitis C virus antibody. Ten patients who underwent gastrectomy during the study period were enrolled. Table 1 shows the characteristics of the patients and lesions. The median tumor size was 38 mm (range: 20 to 70 mm). The clinical T stage was T1 in 1 lesion, T2 in 3 lesions, and T3–4 in 6 lesions. The macroscopic type was 0-IIc in 1 lesion, T2 in 4 lesions, and T3 in 5 lesions.

**Table 1:** Patient and lesion characteristics.

	N=10
Sex	
Male	7
Female	3
Age, median (range), year	73 (68 to 83)
Pathological tumor size, median (range) (mm)	38 (20 to 70)
Macroscopic type	
0-IIc	2
Type2	3
Type3	5
Pathological tumor depth	
pT1b (SM)	2
pT2 (MP)	4
pT3 (SS)	3
pT4a (SE)	1
Histological type	
tub1, tub2	3
por, sig	0
Mixed	7
Unexposed area	
Present	6
Absent	4

Note: SM, submucosa; MP, muscularis propria; SS, subserosa; SE, serosa exposed; tub1, well differentiated adenocarcinoma; tub2, moderately differentiated adenocarcinoma; por, poorly differentiated adenocarcinoma; sig, signet-ring cell carcinoma.

**Fig. 1:**
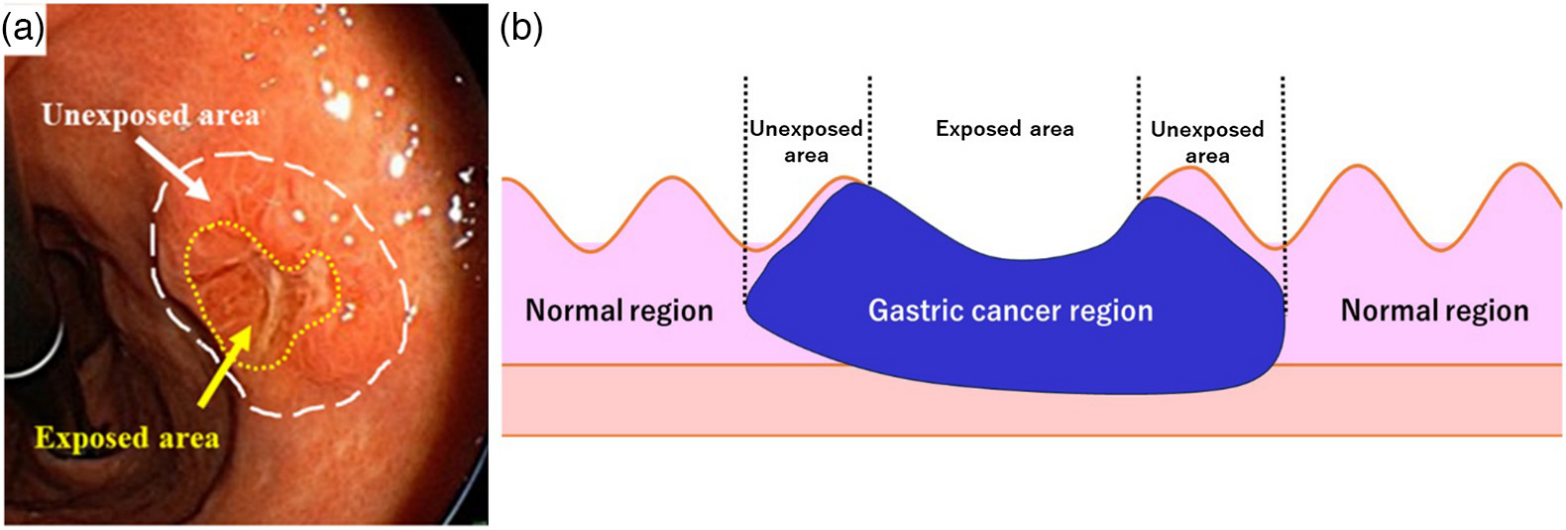
(a) Endoscopic view of gastric cancer. White line is the predicted unexposed area. Yellow line is the predicted exposed area. (b) Histopathology image schema. Cancerous and normal areas were distinguished by the pathologist. Cancerous areas were divided into exposed and unexposed areas, according to their definitions.

The indicators for the types of gastrectomy and a proximal resection margin were determined based on the preference of the surgeon and were primarily based on the Japanese GC treatment guidelines.^6^ All of the surgical specimens were fixed using formalin, cut into 5- or 10 mm-thick slices, stained with hematoxylin and eosin, and evaluated by experienced pathologists. Clinical and pathological outcomes included the Borrmann classification of the tumor, tumor size, depth of invasion, tumor thickness, and histology based on differentiation. The extent of the GC was evaluated separately for exposed and unexposed areas. The exposed area was defined as an area where the cancer itself was pathologically exposed on the surface and not covered by any normal tissue. The non-exposed area was defined as an area where the cancer was present although not exposed on the surface and the surface was covered by normal tissue. The normal areas were defined as areas where the cancer was not present, as shown in Fig. 1.

This study was approved by the Institutional Review Board of the National Cancer Center, Japan (Approval No. 2015-339), which conformed to the provisions of the Declaration of Helsinki and Epidemiological Study Guidelines issued by the Japan Ministry of Health, Labor, and Welfare. All patients provided a written informed consent prior to their inclusion in the study.

### 2.2 Near-Infrared Hyperspectral Image Capture and Data Preprocessing

An imaging system with a high-speed NIR hyperspectral camera (CompoVision, CV-N800HS; Sumitomo Electric Industries, Ltd., Osaka, Japan) was used to obtain the NIR-HSI images (wavelength: 1000 to 2350 nm, wavelength resolution: 6.3 nm, and depth resolution: 14 bit). The detector (NIR spectroscopic camera) captured the data values for each wavelength band on each pixel per line of the image in one scan. Three-dimensional HSI images (x–y–λ axes) were obtained by scanning multiple lines (by sliding the sample stage), thereby producing a virtual “data cube” for processing and analysis.^17^ Each fresh specimen that, prior to the formalin fixation was resected from the stomach, was placed on the sliding stage without trimming. Each NIR-HSI image was acquired once from the mucosal side under illumination from a halogen lamp ($0.96\;W/{\mathrm{cm}}^{2}$). To analyze the HSI data, it is necessary to calibrate the images using dark noise and a white standard for each pixel(i,j). Each reflectance is expressed as follows:

$$R(i,j)=\frac{I_{r}(i,j)-I_{d}(i,j)}{I_{w}(i,j)-I_{d}(i,j)},$$ (1)

where R(i,j) is a row vector of the reflectance spectrum of the obtained image and $I_{r}(i,j)$, $I_{w}(i,j)$, and $I_{d}(i,j)$ are the row vectors of raw, white standard, and dark noise data, respectively. Spectra with wavelengths >1400  nm were removed from the analysis because of the high absorption by the water in those bands and the lower sensitivity of the NIR camera, unlike the transmission spectroscopy.^20^ Additionally, the reflectance rates of more than 70% as highlights and below 10% as shadows were defined in the 1300-nm spectra. Further, the pixels of the necrosis were removed from the dataset and analysis. Normal, exposed, and unexposed GCs were marked by a pathologist to create three regions showing the pixels of the boundary lines. The 5-pixel margin around the boundary lines was excluded while extracting the spectra from each region because the boundary line was drawn freehand by the pathologist. Hence, it was susceptible to error.

NIR spectral measurements reported the variance and the non-specific scattering at the surface of the sample.^22^ The standard normal variate (SNV) of absorbance was used for the baseline correction of the spectrum to reduce the variance and is as expressed as follows:

$$A(i,j)=-\log_{10}(\frac{I_{r}(i,j)-I_{d}(i,j)}{I_{w}(i,j)-I_{d}(i,j)}),$$ (2)

$$Z(x)=\frac{x-\text{mean}(x)}{\mathrm{std}(x)},$$ (3)

where A(i,j) is a row vector of the absorbance spectrum, x is a row vector containing the original spectrum, mean(x) is the mean of x, std(x) is the standard deviation of x, and Z(x) is the SNV-transformed spectrum.

### 2.3 Spectral Data Analysis

A support vector machine (SVM) was used to perform a two-class classification of the normal tissues and entire GC lesions, excluding the 5-pixel margin of the boundary line and necrosis area [inside of the pink line in Fig. 3(B)].

During this process, the pixels to be trained were randomly extracted from a specimen to avoid overfitting. The number of pixels was aligned with normal 200 px and GC 200 px from the confirmed area by pathology. Further, leave-one-out cross-validation was employed, wherein a specimen is classified by a training dataset (each 200 px of normal and GC from nine specimens; total 3600 spectra) that excludes the specimen pixels because the procedure provides an approximately unbiased estimate of the generalization ability.^23^ This algorithm solves an optimization problem expressed as follows:^24^

$$\text{minimize }t(w_{n},\xi_{i})=\frac{1}{2}\overset{k}{\underset{n=1}{\sum }}{\left\|w_{n}\right\|}^{2}+\frac{C}{m}\overset{m}{\underset{i=1}{\sum }}\xi_{i},\;\text{subject to }\langle x_{i},w_{y_{i}}\rangle -\langle x_{i},w_{n}\rangle \geq b_{i}^{n}-\xi_{i}\;(i=1,\ldots ,m),$$ (4)

$$b_{i}^{n}=1-\delta_{y_{i},n},$$ (5)

where $w_{n}$ is a weight vector, C is the cost, and $\xi_{i}$ is the slack variable. Further, the decision function is expressed as

$$g\;\max_{m=1,\ldots ,k}\;\langle x_{i},w_{n}\rangle .$$ (6)

The optimization problem was solved using the decomposition method.^25^ This study used C=1, and the RBF kernel is expressed as

$$K(x_{i},x_{j})=\exp(-\frac{{\left\|x_{i}-x_{j}\right\|}^{2}}{\sigma^{2}}),$$ (7)

where the optimal values of the hyperparameter $\sigma^{2}$ are estimated as follows:

$$\sigma^{2}≔\text{median}\{{\left\|x_{i}-x_{j}\right\|}^{2}|i<j|\}.$$ (8)

The SNV-transformed spectra of the normal tissues, except for the test sample, were averaged. Further, the mean SNV-transformed spectra of the exposed and unexposed regions in the tumor of the test sample were calculated. Hence, the difference in the spectra was obtained by comparing the spectra of the normal and tumor tissues.

### 2.4 Calculation of Prediction Accuracy

The coordinates of the classification pixels were compared to the line drawing images by a pathologist to evaluate the classification accuracy. The area of analysis in this study was limited to the pathologically confirmed area (green area) because only the green area in Fig. 4 is the pathologically evaluated area. This is because the pathologically evaluated sites are the cancer and surrounding normal tissue. The pixels were classified into four groups: GC is predicted as GC [true positive (TP)], GC is predicted as normal [false negative (FN)], normal is predicted as GC [false positive (FP)], and normal is predicted as normal [true negative (TN)]. The false-positive rate and false-negative rate were calculated from the classified pixels. The specificity, sensitivity, and accuracy were expressed as follows:

$$\text{specificity }(\%)=\frac{\mathrm{TN}}{\mathrm{FP}+\mathrm{TN}}\times 100,$$ (9)

$$\text{sensitivity }(\%)=\frac{\mathrm{TP}}{\mathrm{TP}+\mathrm{FN}}\times 100,$$ (10)

$$\text{accuracy }(\%)=\frac{\mathrm{TP}+\mathrm{TN}}{\mathrm{TP}+\mathrm{TN}+\mathrm{FP}+\mathrm{FN}}\times 100.$$ (11)

### 2.5 Comparison of Histopathologic Tumor Thickness

Histopathological tumor thicknesses were compared to determine the factors that contributed to the identified areas (TP pixels) and unidentified areas (FN pixels) of the GC using NIR-HSI. The criteria for the identified and unidentified areas of each specimen were as follows. If the sensitivity was >80%, the pathological sections in the identified areas could be accurately recognized, but the unidentified areas were difficult to recognize. Therefore, tumor thickness was measured only in the identified areas. However, when the sensitivity was <80%, it was possible to recognize pathological sections in the identified and unidentified areas; therefore, tumor thickness was measured in the identified and unidentified areas.

## 3 Results

### 3.1 Endoscopic Images

**Fig. 2:**
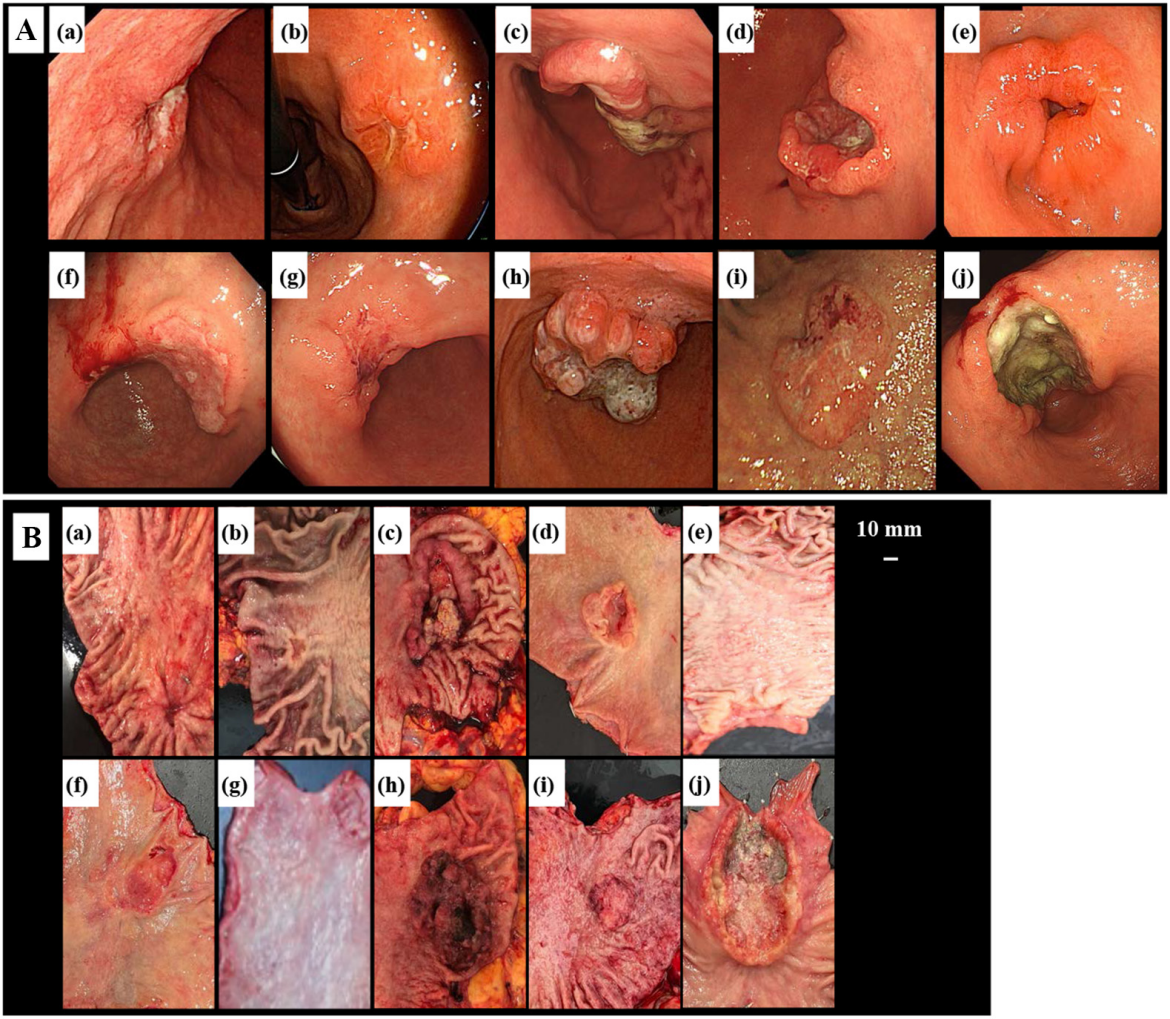
(A), (a)–(j) Endoscopic view of GC. (B), (a)–(j) Visible light photograph of specimens captured using a digital camera.

The endoscopic images of the lesions and pictures of the 10 excised specimens are shown in Figs. 2(A) and 2(B), respectively. Four specimens [d, e, g, and i in Fig. 2(B)] were exposed to the mucosal surface of all regions for cancer. Six specimens [a–c, f, h, and i in Fig. 2(B)] had unexposed cancer in some regions.

**Fig. 3:**
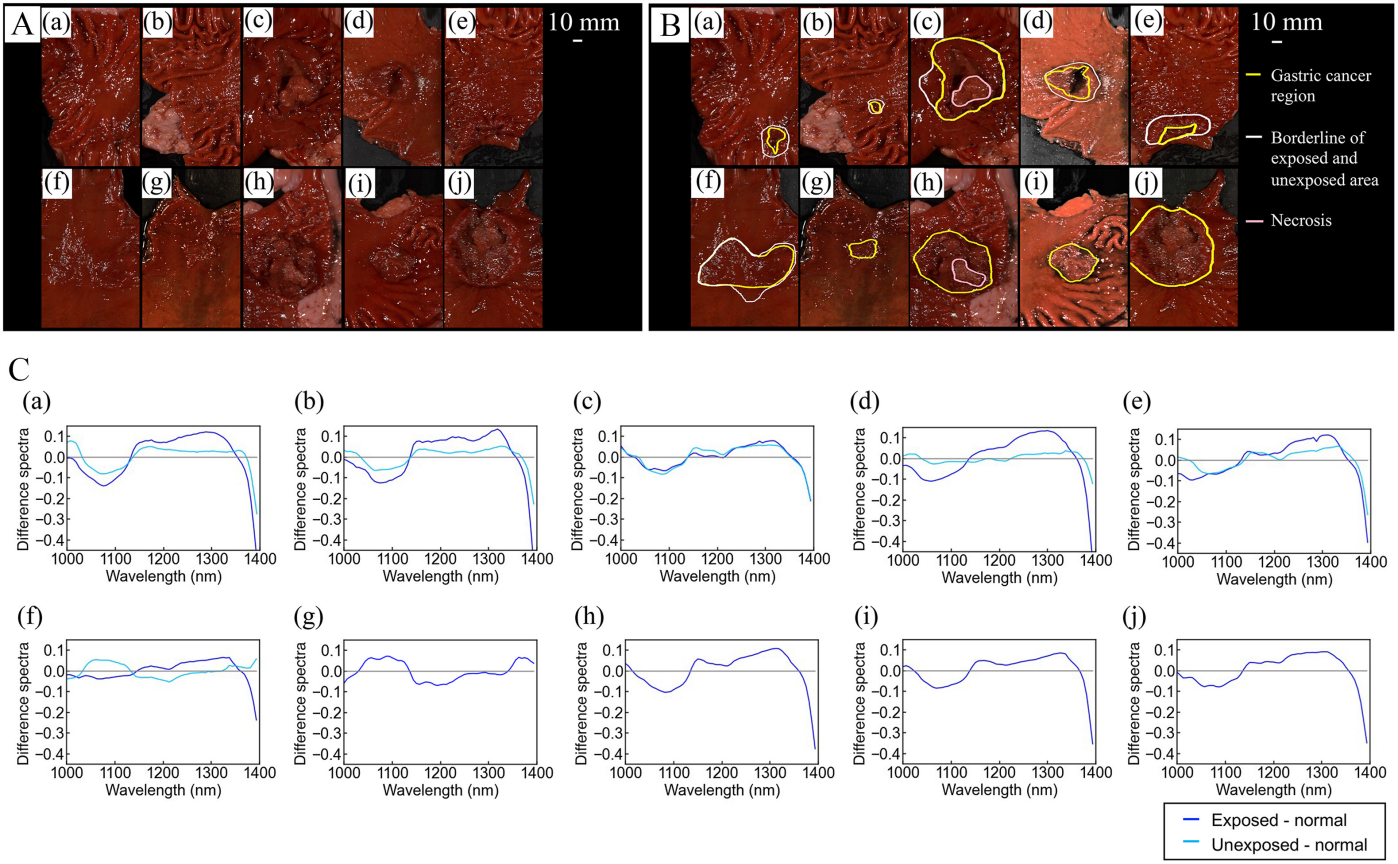
(A), (a)–(j) Pseudocolored pictures of the specimens captured using a CompoVision (NIR camera, R: 1065 nm, G: 1280 nm, B: 1981 nm). (B), (a)–(j) Gastric cancer area (white line), exposed and unexposed areas of the GC (yellow line), and necrosis area (pink line) marked by a pathologist. (C), (a)–(j) The difference in the spectra between the SNV-transformed spectra of normal tissues (leave-one-out training data) and mean SNV-transformed spectra of the exposed and unexposed regions in the tumor of the test sample.

Figure 3(A) shows the pictures of the 10 specimens captured using the NIR-HSI. The GC and normal regions in the NIR-HSI images were marked by a pathologist [Fig. 3(B)]. The white lines represent the extent of the GC, and the yellow ones represent the borderline between the exposed and unexposed areas of the GC. To visualize the trend of the spectral regions contributing to the cancer classification, the difference between the SNV-transformed spectra of the normal tissues (leave-one-out training data) and mean SNV-transformed spectra of the exposed and unexposed regions in the tumor of the test sample is shown in Fig. 3(C).

The results showed that the absorbance difference in the wavelength range of 1050 to 1100 nm and 1380 to 1400 nm was negative and in the wavelength range of 1250 to 1350 nm was positive, except for the unexposed and exposed areas of the specimens (f and g, respectively).

### 3.2 Identification Results of Gastric Cancer and Normal Regions using NIR-HSI

**Fig. 4:**
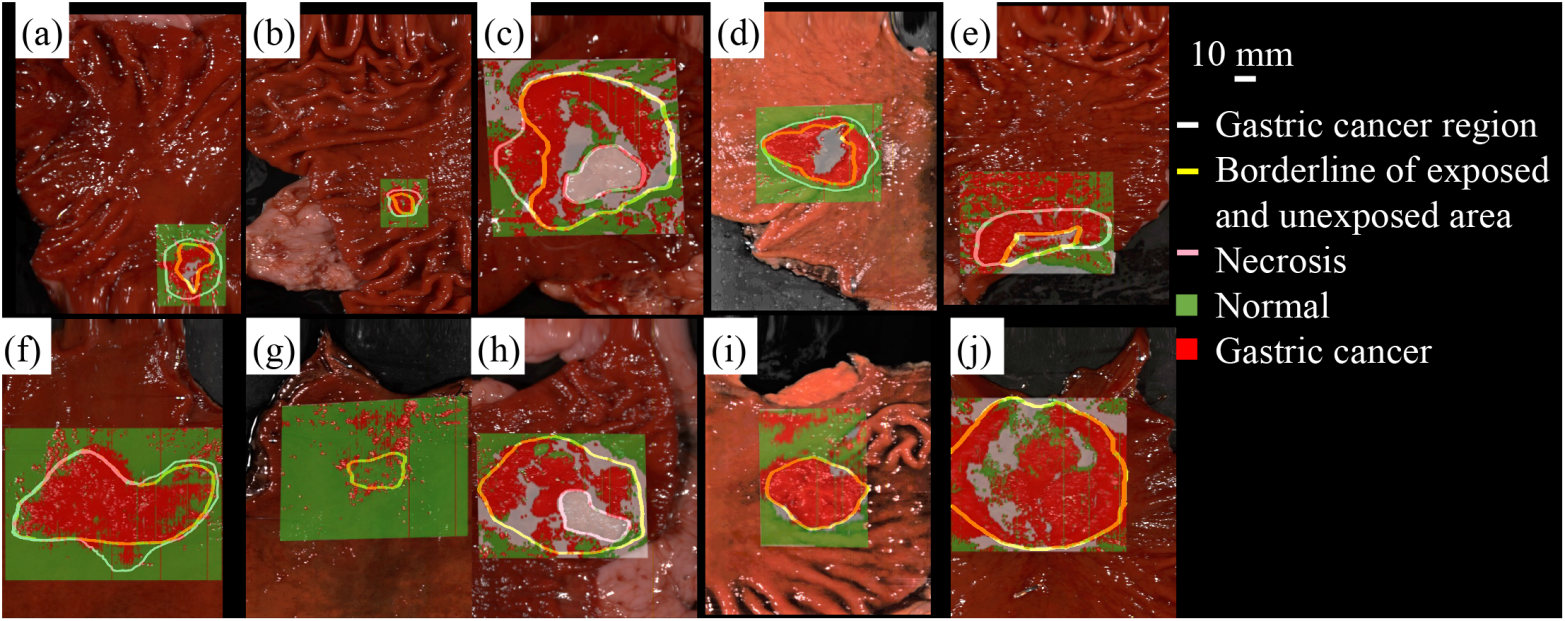
(a)–(j) Gastric cancer region prediction analyzed using machine learning. The color-coded pixels represent the prediction of GC (red) and normal (green).

The analysis of the spectra from the HSI images was performed based on the training data using the SVM algorithm. Further, the GC and normal regions were identified. The pixels that were predicted as GC were assigned a red color, and the normal tissues were assigned a green color. The color-coded pixels that were predicted as GC and normal tissues are shown in the upper images in Figs. 4(a)–4(j). The lower images in Figs. 4(a)–4(j) were merged to include the boundary line of the pathologist. Furthermore, the pixel areas used for calculating the prediction accuracy were also shown. The pixels (459,688 px) were classified as follows: TP: 160,693 px, FN: 35,517 px, FP: 55,567 px, and TN: 207,911 px. The specificity, sensitivity, and accuracy of 74.8%, 77.2%, and 79.7%, respectively, were evaluated from the classified pixels.

Table 2 presents the results. The prediction results of the exposed area were classified as follows: TP: 138,545 px and FN: 27,971 px. The sensitivity of 79.7% was calculated from the classified pixels. Additionally, the prediction results for the unexposed area were classified as follows: TP: 22,148 px and FN: 7545 px. Furthermore, the sensitivity of 68.5% was calculated from the classified pixels. The complete results are presented in Table 3.

**Table 2:** Prediction results of the NIR-HSI analysis. The GC region [the inside of the white line of Fig 2(b)] was defined as “positive,” and the outside of the GC was defined “negative” in the pathologically confirmed area. The 5 px margin of the white line and necrosis area were excluded from the analysis.

No.	TP (pixel)	FN (pixel)	FP (pixel)	TN (pixel)	Specificity (%)	Sensitivity (%)	Accuracy (%)
(a)	4070	2041	1310	7607	85.3	66.6	77.7
(b)	1520	379	777	3891	83.4	80.0	82.4
(c)	30,443	5506	7007	19,267	73.3	84.7	79.9
(d)	10,952	1804	1075	17,886	94.3	85.9	90.9
(e)	10,095	1387	15712	11,450	42.2	87.9	55.8
(f)	31,940	10354	6487	49,284	88.4	75.5	82.8
(g)	1153	3326	7624	64,705	89.5	25.7	85.7
(h)	17,642	3438	2143	10,518	83.1	83.7	83.5
(i)	13,460	485	6760	19,821	74.6	96.5	82.1
(j)	39,418	6797	6672	3482	34.3	85.3	76.1
Total	160,693	35,517	55,567	207,911	74.8 ± 19.3	77.2 ± 18.7	79.7 ± 8.9

Note: TP, true positive; FN, false negative; FP, false positive; and TN, true negative.

**Table 3:** Prediction results in the exposed and unexposed areas during the NIR-HSI analysis. Among the pixels analyzed in Table 2, the TP and FN of the area inside the yellow line in Fig. 3(B) were defined as “exposed,” and the TP and FN of the area between the white and yellow lines were defined as “unexposed”.

No.	Exposed			Unexposed		
	TP (pixel)	FN (pixel)	Sensitivity (%)	TP (pixel)	FN (pixel)	Sensitivity (%)
(a)	2096	300	87.5	1974	1741	53.1
(b)	901	121	88.2	619	258	70.6
(c)	24236	4855	83.3	6207	651	90.5
(d)	7816	414	95.0	3136	1390	69.3
(e)	1387	535	72.2	8708	852	91.1
(f)	30436	7701	79.8	1504	2653	36.2
(g)	1153	3326	25.7	—	—	—
(h)	17,642	3438	83.7	—	—	—
(i)	13,460	485	96.5	—	—	—
(j)	39,418	6797	85.3	—	—	—
Total	138,545	27,971	79.7 ± 19.1	22,148	7545	68.5 ± 19.5

Note: TP, true positive; FN, false negative.

### 3.3 Relationship between Tumor Thickness and Identifiable Area using NIR-HSI

**Fig. 5:**
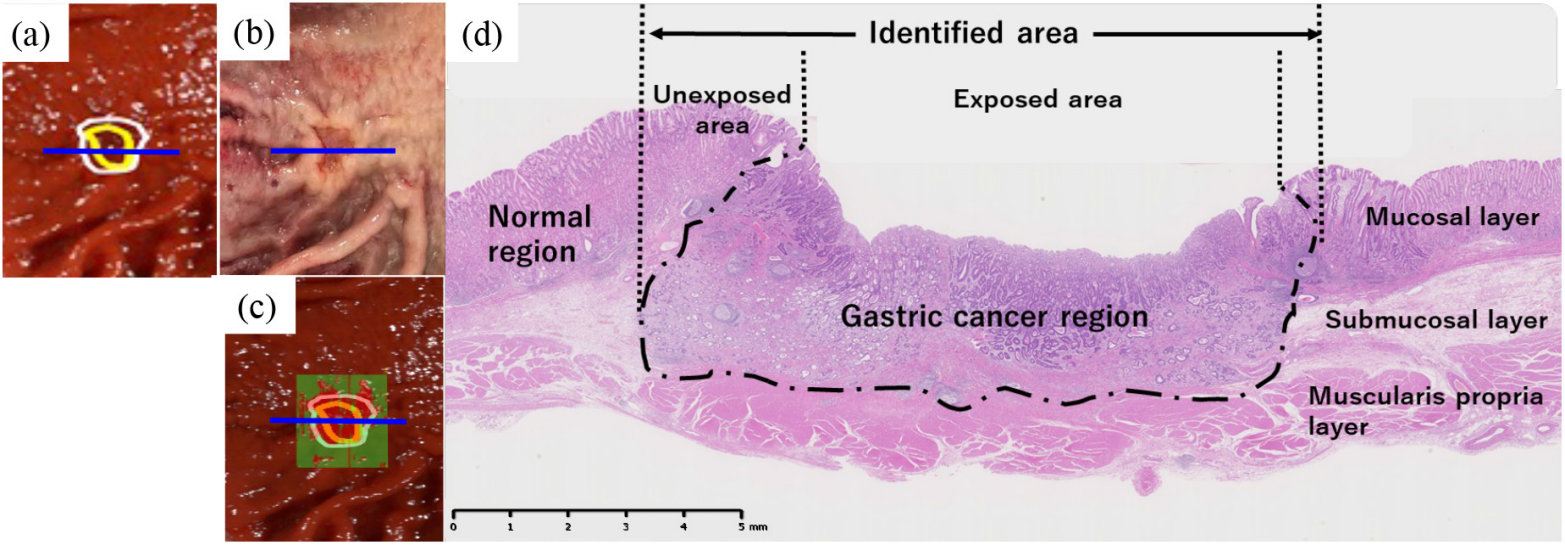
For the case in Fig. 2A(b), (a) NIR-HSI image showing the gastric cancer region (white line) and borderline between the exposed and unexposed area (red line), (b) resected specimen after fixation, and (c) image showing the predicted GC and normal tissue, colored red and green, respectively. (d) The histological image revealing the area of the cut along the blue line in (a)–(c). The histology from the surgical resection revealed adenocarcinoma in the deep submucosal deep layer. The GC was identified using NIR-HSI regardless of the exposed or unexposed area.

Table 4 shows the tumor thickness in the GC region. NIR hyperspectral imaging detected cancer areas with pathologic thicknesses of >2  mm regardless of whether the cancer was exposed on the mucosal surface or not (Fig. 5).

**Table 4:** Relationship between the tumor thickness and identifiable area using NIR-HSI.

	(a)	(b)	(c)	(d)	(e)	(f)	(g)	(h)	(i)	(j)
Tumor thickness, range (mm)										
Identifiable area	2.4 to 8.4	2.0 to 4.0	4.2 to 16.0	2.2 to 11.4	2.2 to 5.5	2.1 to 10.4	—	4.8 to 17.4	3.2 to 4.8	3.4 to 13.0
Unidentifiable area	0.2 to 1.3	0.6 to 1.4	—	1.2 to 1.4	1.3 to 1.5	0.6 to 1.2	0.4 to 1.3	—	—	—

**Fig. 6:**
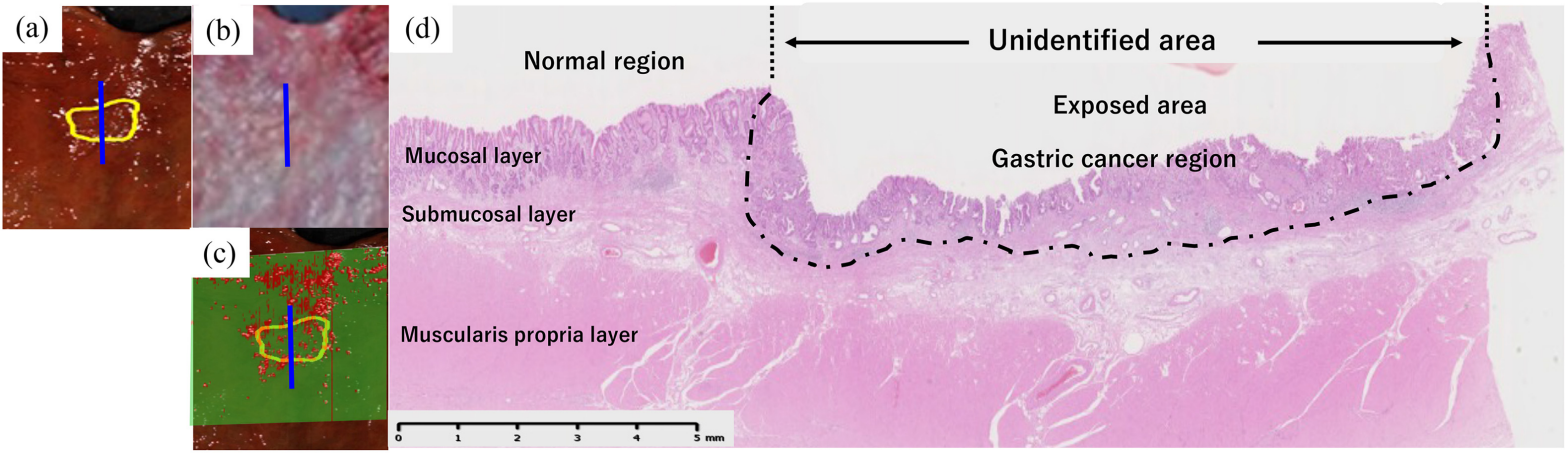
For the case in Fig. 2A(g), (a) NIR-HSI image marked with GC, (b) resected specimen after fixation, and (c) image showing the predicted GC and normal tissue, colored red and green, respectively. (d) The histological image revealing the area of the cut along the blue line in (a)–(c). The histology from the surgical resection revealed adenocarcinoma in the submucosal slight layer. The GC was unidentified using NIR-HSI.

However, thin-tumor areas, including those exposed on the mucosal surface, were unidentified as cancerous (Fig. 6).

Additionally, in the prediction results of the NIR-HSI analysis as shown in Table 3, specimens g and f had the lowest sensitivity in the exposed and unexposed regions, respectively. As shown in Fig. 3(C), the spectral trends of both specimens were different from those of the other highly sensitive specimens. This may be because the lesion tissue was thin (Table 4), whereas the molecular structures of the samples were different from those of the other samples.

## 4 Discussion

This study evaluated the extent of the GC, including the unexposed areas that were difficult to recognize under white light or by machine learning using the NIR-HSI data. The findings of this study are novel because previous NIR-HSI studies on GC did not discuss the identification of cancerous areas under normal mucosa.

This study identified the exposed area of the GC as cancerous with high sensitivity, that is, with 79.7% and 68.5% sensitivities in the exposed and unexposed areas, respectively. However, the overall sensitivity was lower in the unexposed area than in the exposed area. Further, several FPs were detected in thin cancerous areas, which is possibly owing to the characteristics of the NIR absorption spectroscopy that uses reflected light: (i) the thicker the normal mucosa covering cancer, the more attenuated the characteristic absorption spectrum of cancer and (ii) the thinner the cancer, the sparser the information in the absorption spectrum. As shown in Fig. 3(C), the absorbance difference between the normal area spectra in the training data and that of the unexposed area was smaller than that of the exposed area. Areas with tumor thicknesses of >2  mm were identified using the NIR-HSI, even in unexposed areas (Table 4). However, areas with thin tumors remained unidentified in either the exposed or unexposed areas. This study shows that NIR-HSI can be used to accurately recognize unexposed areas that are difficult to recognize under white light when the thickness is 2 mm or more.

This study was limited by the small number of specimens. However, it classified NIR-HSI-captured pixels (total pixel: 459,688 px) using the leave-one-out cross-validation procedure. Hence, the sample size was sufficient, and its generalizability was demonstrated. The results for shallow-depth areas were inadequate because numerous specimens in the study had advanced cancer, and sufficient data on the areas with shallow depths of cancer invasion were lacking. Gathering more data on shallow lesions was necessary, as represented by the specimen in Fig. 2(B) [case (g)]. Hence, the shallow lesions were unidentified as cancers. The optimization of the measurement conditions, such as changes in the illumination method of the NIR-HSI system, specimen placement, and design focal length, improved the detection performance. Additionally, although an SVM was adopted as a machine learning method in this study, other methods such as neural network and principal correlation analysis have been proposed to identify lesions. The identification accuracy may be improved by investigating the optimal algorithm and parameter. Moreover, significantly increasing the number of specimens would broaden the range of options for popular deep learning techniques and allow for the exploration of more robust classifiers.

However, imaging conditions in the stomach using an endoscope with NIR-HSI differed from those of our study. For example, the postoperative specimen was imaged from the front in this study, though it may not be possible in the case of an endoscope in the stomach. Additionally, the absorption spectra may change before gastrectomy because of the blood flow and other factors. Hence, the NIR-HSI devices for endoscopic use must be developed, and the optimization of the measurement conditions must be considered in the future.

Our group is currently developing an NIR-HSI device that can be used with endoscopes. We expect to perform an endoscopic NIR-HSI analysis in the near future. If this technology is used with an endoscope, it may be possible to intraoperatively or preoperatively identify the extent of the unexposed area and define a more appropriate line for the resection of the stomach.

## 5 Conclusion

This study accurately identified the exposed and unexposed GC areas with thicknesses >2  mm. In the future, we expect to develop an NIR-HSI system that will be used with endoscopes to enhance its practicality.

## Data Availability

The datasets generated and analyzed during the current study are not publicly available owing to containing personal patient information but are available from the corresponding author on reasonable request.
